# Necrobiosis and T-lymphocyte infiltration in retrieved aseptically loosened metal-on-polyethylene arthroplasties

**DOI:** 10.3109/17453674.2011.625534

**Published:** 2011-11-24

**Authors:** Christoph von Domarus, Jens P Rosenberg, Wolfgang Rüther, Jozef Zustin

**Affiliations:** ^1^Department of Orthopaedics; ^2^Institute of Pathology, University Medical Center Hamburg-Eppendorf, Hamburg, Germany

## Abstract

**Background and purpose:**

Soft tissue necrobiosis and T-lymphocyte infiltration within the periprosthetic soft tissue have been linked to a suggested hypersensitivity reaction of the delayed-type following the metal-on-metal arthroplasty. While we observed both synovial necrobiosis and lymphocyte infiltrates in synovial tissues with failed metal-on-polyethylene prostheses, we hypothesized that both findings are unspecific for metal-on-metal bearing coupes. Thus, we wished to quantify the extent of necrobiosis and the amount of T-lymphocyte infiltration in aseptically loosened metal-on-polyethylene prostheses.

**Materials and methods:**

We analyzed 28 consecutive synovial biopsy specimens obtained at revision surgery of aseptically loosened metal-on-polyethylene prostheses (19 hips and 9 knees) and quantified both the extent of necrobiosis vertically and the density of CD3+, CD4+, and CD8+ lymphocytes within the joint capsular tissue. We excluded patients with inflammatory skeletal disease or with a history of metal hypersensitivity.

**Results:**

We found necrobiosis in 23 of 28 cases and it was most often connected with the superficial portions of the synovium. Necrobiosis of deeper tissues was seen in 8 specimens and it was strongly associated with superficial necrobiosis. While CD3+ lymphocytes were detected in each biopsy, 4 cases with more than 300 CD3+ lymphocytes were identified in the group of 26 cases that presented with more than 100 CD3+ lymphocytes within one high-power field. 16 cases with more than 100 CD3+ lymphocytes also showed concomitant superficial necrobiosis of the synovium. In the inflammatory infiltration of periprosthetic synovium, CD8+ lymphocytes predominated over CD4+ cells.

**Interpretation:**

Synovial necrobiosis and infiltration of T-lymphocytes are common findings in tissues around aseptically loosened metal-on-polyethylene arthroplasty in patients without a clinically suspected metal hypersensitivity reaction. Thus, neither necrobiosis nor infiltration of T-lymphocytes should be considered to be specific for failed metal-on-metal bearings or metal hypersensitivity reaction.

The re-introduction of metal-on-metal (M-o-M) bearings has been associated with adverse biological reactions that appear to be related to metal hypersensitivity reactions. Solid periprosthetic masses showing necrotic granulomatous pseudotumors (Pandit et al. 2008a, Mahendra et al. 2009), necrobiosis (Doorn et al. 1996, Pandit et al. 2008b, Mahendra et al. 2009, Campbell et al. 2010), enlarged bursae (Fang et al. 2008), and inflammatory infiltrates of mainly T-lymphocytes (Willert et al. 2005, Pandit et al. 2008a, Mahendra et al. 2009, Thomas et al. 2009, Zustin et al. 2009) have been observed in periarticular tissues from failed M-o-M prostheses and have been suggested to be characteristic of delayed-type hypersensitivity reactions to metal. While specific laboratory or clinical tests for arthroplasty-related hypersensitivity reactions are currently unavailable, both necrobiosis (Willert et al. 2005, Aroukatos et al. 2010) and infiltration of lymphocytes (Willert et al. 2005) have also been observed in cases involving bearing couples other than M-o-M. Similarly, we have observed necrobiosis and lymphocyte infiltrations within periprosthetic synovial tissues and do not consider them to be specific for metal hypersensitivity. We hypothesized that necrobiosis and T-lymphocyte infiltration are not specific to synovial changes in periprosthetic synovium of M-o-M prostheses.

To determine how often the histopathological findings suggestive of the delayed-type hypersensitivity reaction to metal in M-o-M arthroplasty can also be observed in the synovium obtained from joints with aseptically loosened metal-on-polyethylene (M-o-PE) prostheses, we asked: (1) what is the frequency and morphological pattern of necrobiosis in these cases, and (2) what is the prevalence and number of periarticular soft tissue T-lymphocytes in these specimens?

## Patients and methods

We retrospectively analyzed 28 consecutive synovial biopsies obtained at revision surgery for aseptic loosening of M-o-PE arthroplasties operated by the senior author (WR) from January to June 2008. All implantations had been performed for primary osteoarthritis. We excluded patients with a history of metal hypersensitivity (contact dermatitis) and/or rheumatoid disease. 11 of the patients were men (median age: 69 years; IQR: 66–73) and 17 were women (median age: 71 years; IQR: 63–74). 19 specimens were from the hip (median duration of implantation: 120 months; IQR: 77–174), and 9 were from the knee (median duration of implantation: 51 months; IQR: 20 to 156). All procedures were unilateral. Infection was not suspected, either clinically (negative radiographic and laboratory findings) or microscopically (negative finding of neutrophilic polymorphonuclear leukocytes in the biopsy specimen).

Immediately after the excision biopsy of synovial tissue, the specimens were fixed in formalin and sent to the laboratory for histopathological analysis. They were analyzed macroscopically, photographed, cut in a plane vertical to the synovial surface, and embedded in paraffin wax. The histopathological slides were stained using the hematoxylin and eosin, elastica van Gieson, and chloracetate esterase staining methods. All specimens were also analyzed under polarized light. Immunohistochemical staining was performed simultaneously with monoclonal mouse anti-human antibody against the T-lymphocyte marker CD3 (clone F7.2.38; Dako M7254; 1:25 dilution), CD4 (clone 1F6; Biocare Medical; 1:25) and CD8 (clone anti-CD8; Dako M7103; 1:100). The immunohistochemical analysis was validated using positive controls (tonsil) and negative controls (by omitting the primary antibody).

Necrobiosis was defined as regressive soft tissue change showing the ghost outlines of histiocytes in areas with maintained collagen structure. Under polarized light, birefringent wear particles were apparent within the lesions, often within or adjacent to ghost outlines of formerly visible histiocytes, and/or associated with the loss of former cell outlines. Both histiocytes and fibroblasts showed degenerated or absent nuclei (Doorn et al. 1996). Stained sections were coded and analyzed for the presence of necrobiosis by 2 independent observers (CD and JZ). Weight kappa analysis was used to calculate the reliability of inter-observer agreement for diagnosing necrobiosis. Inter-observer agreement on diagnosis of the presence or absence of necrobiosis was observed in 89% of cases (kappa = 0.70).

**Figure 1.**
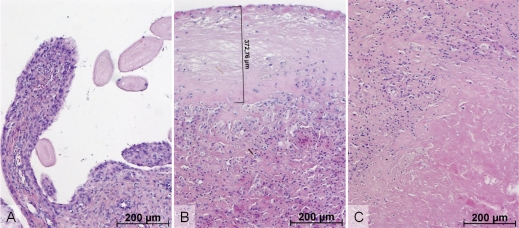
Synovial tissue necrobiosis. (A) Villous proliferations without stainable synoviocytes, blood vessel walls, stromal cells, and inflammatory infiltrates (at top right) were characteristic of villous necrobiosis. (B) Band-like acellular areas of the synovial surface (upper half) showing scattered ghost cells were a typical finding in synovial surface necrobiosis. Their maximal extent vertically was measured digitally and recorded in each case. (C) Similar acellular areas (at lower right) in deeper periarticular soft tissue without any contact with the synovial surface in this specimen were referred to as deep necrobiosis. (Hematoxylin and eosin).

Villous necrobiosis (Figure 1A) was diagnosed in those areas that displayed complete necrobiosis of the villous proliferation on the synovial surface. Superficial necrobiosis (Figure 1B) was characterized by a flat zone of necrobiosis, which was parallel to the surface of the specimen and was in broad contact with it. The most representative area was chosen for subsequent metric analysis. CD and JZ recorded the maximal vertical extent of the necrobiosis using a Zeiss AxioCam MRc camera (Carl Zeiss MicroImaging GmbH, Germany) and AxioVision Release 4.6.3 software (Carl Zeiss Imaging Solutions GmbH, Germany). Deep soft tissue necrobiosis (Figure 1C) was defined as the occurrence of soft tissue necrobiosis without any contact with the luminal articular surface of the specimen in the plane analyzed.

**Figure 2.**
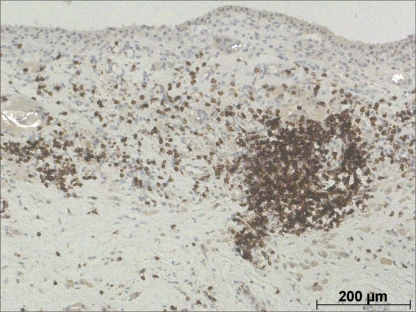
Immunohistochemistry with antibody to CD3 antigen was performed in order to identify T-lymphocytes within the synovial soft tissue. In this particular case, both perivascular (mid right) and diffuse CD3+ lymphocytes were detected. Polyethylene wear particles were apparent under polarized light microscopy in several foreign body giant cells and histiocytes. (Embedding in paraffin wax; microscopic analysis under polarized light; immunohistochemical reaction with CD3 antibody).

To identify and quantify T-lymphocytes, JR and JZ chose areas with maximum immunohistochemically positive CD3, CD4, and CD8 cells and JR counted all positive cells per high-power field (original magnification: ×400; 0.16 mm^2^) (Figure 2).

The results are expressed as median and interquartile range (IQR). For comparison of the nominal variables, Fisher's exact test was used. The depth of necrobiosis and the number of T-lymphocytes were not normally distributed and were analyzed using the non-parametric Mann-Whitney test. Correlations were considered significant when the p-value was less than 0.05. All data were analyzed using SPSS software version 18.0.

## Results

Necrobiosis was detected in 23 of 28 synovial biopsies: 13 specimens showed necrobiosis of the villous synovial projections, 17 cases showed superficial necrobiosis, and 8 biopsies had deep synovial tissue necrobiosis (Table 1). Necrobiosis of the villous synovial projections was found in 6 cases without coexisting superficial necrobiosis (p = 0.4, r = –0.13). However, necrobiosis of deep synovial soft tissue was detected in 8 specimens that all also showed superficial necrobiosis (p = 0.008, r = 0.51). The prevalence of necrobiosis in males (9 of 11 men) was similar to that in females (14 of 17 women; p = 0.7, r = 0.007). Similarly, the depth of necrobiosis in males (median extent of necrobiosis vertically: 209 µm; IQR: 0–443) was not substantially different from that in females (median extent of necrobiosis vertically: 327 µm; IQR: 0–1,327; p = 0.3). Moreover, there was no difference in the extent of necrobiosis between failed hips (median extent of necrobiosis vertically: 327 µm; IQR: 0–982) and failed knees (median extent of necrobiosis vertically: 0 µm; IQR: 0–841; p = 0.3). In 7 cases, we detected macrophages with few ingested metal wear particles, which could be classified as slate blue histiocytes (Doorn et al. 1996). Metallosis was present in 4 of 19 retrieved hips and in 3 of 9 aseptically loosened knee prostheses (p = 0.7, r = 0.13). Furthermore, we did not find any relationship between the presence of metallosis and superficial (p = 0.2, r = 0.30), villous (p = 0.7, r = 0.12), or deep necrobiosis (p = 1, r = 0) but the extent of necrobiosis in cases with metallosis (median extent of necrobiosis vertically: 1,163 µm; IQR: 310–1,491) was larger than in cases without microscopically evident metal particles (median extent of necrobiosis vertically: 209 µm; IQR: 0–481; p = 0.04).

**Table 1.** Patterns of necrobiosis in the study cohort (Fisher's exact test)

	Villous necrobiosis	Superficial necrobiosis	Deep necrobiosis
Joint			
Hip (n = 19)	10	13	7
Knee (n = 9)	3	4	1
p-value	0.4	0.4	0.2
r-value	–0.18	–0.23	–0.27
Metal wear			
Metallosis present n = 7	4	6	2
Metal wear invisible n = 21	9	11	6
p-value	0.7	0.2	1.0
r-value	0.12	0.30	0.0

CD3+ lymphocytes were detected in each specimen (median number of CD3+ cells per high-power field (HPF): 176; IQR: 129–260). While none of the patients showed less than 50 CD3+ lymphocytes per HPF and only 2 patients had less than 100 CD3+ lymphocytes per HPF, 4 specimens had more than 300 CD3+ lymphocytes per HPF. 3 of the 4 patients who had more than 300 CD3+ lymphocytes per HPF were men. The number of CD3+ lymphocytes was not statistically significantly different between those cases without superficial necrobiosis (median number of CD3+ lymphocytes per HPF: 203; IQR: 136–277) and cases with positive findings (median number of CD3+ lymphocytes per HPF: 164; IQR: 127–242; p = 0.4). In addition, the number in men (median number of CD3+ lymphocytes per HPF: 185; IQR: 102–363) was similar to that in women (median number of CD3+ lymphocytes per HPF: 166; IQR: 135–249; p = 0.8). One of these 4 biopsy specimens with more than 300 CD3+ lymphocytes per HPF did not show necrobiosis. When compared with failed hips (median number of CD3+ lymphocytes per HPF: 163; IQR: 127–228), soft tissue specimens from knee joints with aseptically loosened M-o-PE arthroplasty did not have a substantially different number of T-lymphocytes (median number of CD3+ lymphocytes per HPF: 193; IQR: 144–274; p = 0.5). It is worthy of mention that superficial necrobiosis was detected histopathologically in 16 cases that also presented with more than 100 CD3+ lymphocytes. Finally, we did not find any significant differences in the number of lymphocytes in the cases with metallosis (median number of CD3+ lymphocytes per HPF: 185; IQR: 134–270) compared to the cases with negative findings (median number of CD3+ lymphocytes per HPF: 166; IQR: 127–253; p = 0.9) (Table 2).

**Table 2.** Density of T-lymphocyte subpopulations in the study cohort (Mann-Whitney U test). Values are median (IQR)

	CD3+ lymphocytes	CD4+ lymphocytes	CD8+ lymphocytes
Joint			
Hip (n = 19)	163 (127–228)	41 (36–55)	123 (67–157)
Knee (n = 9)	193 (144–274)	55 (35–69)	114 (106–172)
p-value	0.5	0.3	0.3
Metal wear			
Metallosis present (n = 7)	185 (134–270)	49 (31–59)	103 (67–157)
Metal wear invisible (n = 21)	166 (127–253)	41 (37–61)	127 (87–168)
p-value	0.9	0.8	0.7
Necrobiosis			
Necrobiosis present (n = 23)	166 (127–270)	42 (36–59)	114 (67–157)
Necrobiosis absent (n = 5)	203 (128–306)	51 (39–67)	151 (93–230)
p-value	0.7	0.4	0.4

The infiltration of T-lymphocytes involved both CD4+ and CD8+ cells, which were detectable immunohistochemically in every specimen. We did not find any statistically significant differences between CD4+ and CD8+ T-lymphocyte counts (separately) according to anatomic location, whether or not there was metallosis, or whether or not there was necrobiosis (Table 2).

## Discussion

Although necrobiosis and T-lymphocyte infiltration have been observed in failed M-o-M cases suggestive of delayed-type hypersensitivity to metal, the specificity of such findings has been considered to be uncertain. In fact, most reports on this issue described single cases or small groups of cases, and seldom considered control tissues from non-M-o-M failures. In our routinely diagnosed cases, we have observed both necrobiosis and lymphocyte infiltration of periprosthetic synovial soft tissues. Thus, we wanted to investigate the morphological patterns of necrobiosis and the amount of T-lymphocyte infiltration in retrieved M-o-PE prostheses revised for aseptic loosening and we conducted the current retrospective observational morphological study.

### Necrobiosis of the periprosthetic synovial tissue

We observed necrobiosis in the majority of study cases. Although the diagnosis of necrobiosis did not appear to be problematic within villous projections or deeply localized lesions, it was more of a problem in a few cases within the areas with fibrinous effusion attached to the synovial surface that were also associated with the loss of synoviocytes. In these cases, staining with elastica van Gieson was helpful for differentiation between fibrinous exudates and necrotic tissue. While all cases with deep soft tissue necrobiosis also showed superficial necrobiosis, we hypothesized that such areas might represent folding of the synovial surface with large or irregularly deep invading superficial necrobiosis promoted by local anatomic variations in the structure and elasticity of the adjacent soft tissue. One can only speculate whether such propagation of superficial necrobiosis into deeper soft tissue also plays a substantial role in the pathogenesis of the anterior localized soft tissue granulomatous pseudotumors (Pandit et al. 2008a, b) following M-o-M hip arthroplasty; however, the histopathological findings appear to be essentially identical (Fujishiro et al. 2011). In our study cohort of synovial biopsies from aseptically loosened M-o-PE prostheses, there was no relationship between metallosis and the prevalence of necrobiosis, but the extent of necrobiosis vertically was greater in cases with metallosis.

### Lymphocyte infiltration of the periprosthetic soft tissue

Although lymphocyte infiltration of periprosthetic tissues was related to adverse biological reactions following M-o-M arthroplasty, there is an increasing amount of data on the prevalence of lymphocyte infiltration in non-M-o-M failures. Willert et al. (2005) observed lymphocyte infiltrates in 4 out of 11 M-o-PE control group cases. Aroukatos et al. (2010) found T-lymphocytes within scarce inflammatory infiltrate of the neocapsule in failed ceramic-on-polyethylene prostheses. These authors considered more than 50 lymphocytes per HPF as being excessive infiltration (Doorn et al. 1996, Aroukatos et al. 2010) and Willert et al. (2005) described more than 100 lymphocytes per HPF in diffuse inflammatory infiltrate or 10 perivascular lymphocyte aggregations as being excessive when investigated under low power (original magnification: ×4). Recently, Fujishiro et al. (2011) reported perivascular lymphocyte infiltration in 53% and diffuse lymphocyte infiltration in 62% of 107 patients with aseptic loosening. In contrast to the former study, we did not find any relationship between metallosis and the extent of CD3+ lymphocyte infiltration into the periprosthetic synovial membrane. Ng et al. (2011) reported a higher frequency of perivascular lymphocyte infiltrates in specimens from revised total knee arthroplasties than in specimens from hip arthroplasties, but with no difference in severity of infiltration. In our cohort, we did not observe any difference in the frequency or density of CD3+ lymphocyte infiltration between the specimens from synovium of aseptically loosened knee prostheses and hip prostheses.

For the purposes of the current study, we did not differentiate between the diffuse and perivascularly located lymphocytes but counted the number of CD3+ lymphocytes per HPF in areas with maximum infiltration in order to overcome the limitations of descriptive grading systems. Based on our results, it seems probable that a cut-off at 100 lymphocytes per HPF is a relatively low value for consideration of the lymphocyte infiltration to be excessive. Indeed, in our earlier study on morphological findings in M-o-M hip resurfacing revised for groin pain (Zustin et al. 2009), we found up to 260 intraosseous lymphocytes per HPF in the control group of patients with hip osteoarthritis at the time of the primary implantation in total hip arthroplasty. Thus, we used an even more conservative criterion of 300 intraosseous lymphocytes per HPF for the definition of excessive lymphocyte infiltration (Zustin et al. 2009, 2010).

Even though we specifically focused on the number of T-lymphocytes, this particular inflammatory cell population was present in all cases. However, even if we speculate that some of the cases were patients with asymptomatic metal hypersensitivity, this result was rather unexpected and we did not find any explanation other than the fact that T-lymphocytes are a well-known to be a player in the periprosthetic cellular immune response in aseptic loosening of M-o-PE arthroplasties in patients with no history of rheumatoid disease or metal hypersensitivity.

### Limitations of our study

We recognize that our study had several limitations. First, because the exact anatomic location of the biopsy could not be assessed retrospectively, we ignored the possible anatomic variability in the layering of the synovial membrane and capsular soft tissue both within each joint and between knee and hip joints. However, it is possible that the extent of necrobiosis and the soft tissue invasion of wear particles are substantially affected by the variable thickness of loose soft tissues between the less elastic fibrous joint capsule and synovial surface. For the same reason, we could not exclude potential entrapment or impingement of the periarticular soft tissue that might also contribute to ischemic changes in the villous synovial projections. Secondly, because it is generally accepted that T-lymphocytes are pathogenetically associated with delayed-type hypersensitivity (Skoglund and Egelrud 1991, Schmid et al. 2006, Mahendra et al. 2009, Thomas et al. 2009, Zustin et al. 2009), we did not analyze the presence and number of B-lymphocytes or other inflammatory cells. Thirdly, we observed metal particles in 7 cases using the light microscope; however, measurements of metal ions or surface wear were not performed to assess the amount of microscopically undetectable metal particles. Although the polyethylene wear particles were clearly identified under polarized light in each specimen, we did not observe massive metal wear macroscopically in any of the study cases. Fourthly, even though patients with a definite history of metal allergy were not included, we cannot rule out the possibility that there were cases with an asymptomatic course of delayed-type hypersensitivity. Recently, Thyssen et al. (2009) analyzed the patch-test database and did not find any increased risk of surgical revision in patients with metal allergies. Moreover, there was no elevated risk of metal allergy in cases that were operated (Thyssen et al. 2009).

### Conclusion

Based on our results, we suggest that both synovial necrobiosis and T-lymphocyte infiltration are common findings in tissues around aseptically loosened metal-on-polyethylene arthroplasty in patients without any clinically suspected metal hypersensitivity reaction. Thus, neither necrobiosis nor T-lymphocyte infiltration should be considered specific for failed metal-on-metal bearings or metal hypersensitivity reaction.
